# Genetic Risk Score Predicting Risk of Rheumatoid Arthritis Phenotypes and Age of Symptom Onset

**DOI:** 10.1371/journal.pone.0024380

**Published:** 2011-09-12

**Authors:** Lori B. Chibnik, Brendan T. Keenan, Jing Cui, Katherine P. Liao, Karen H. Costenbader, Robert M. Plenge, Elizabeth W. Karlson

**Affiliations:** 1 Program in Translational NeuroPsychiatric Genomics, Department of Neurology, Brigham and Women's Hospital, Boston, Massachusetts, United States of America; 2 Section of Clinical Sciences, Division of Rheumatology, Immunology and Allergy, Department of Medicine, Brigham and Women's Hospital, Boston, Massachusetts, United States of America; 3 Division of Rheumatology, Immunology and Allergy, Department of Medicine and Division of Genetics, Brigham and Women's Hospital, Boston, Massachusetts, United States of America; South Texas Veterans Health Care System, United States of America

## Abstract

**Background:**

Cumulative genetic profiles can help identify individuals at high-risk for developing RA. We examined the impact of 39 validated genetic risk alleles on the risk of RA phenotypes characterized by serologic and erosive status.

**Methods/Principal Findings:**

We evaluated single nucleotide polymorphisms at 31 validated RA risk loci and 8 Human Leukocyte Antigen alleles among 542 Caucasian RA cases and 551 Caucasian controls from Nurses' Health Study and Nurses' Health Study II. We created a weighted genetic risk score (GRS) and evaluated it as 7 ordinal groups using logistic regression (adjusting for age and smoking) to assess the relationship between GRS group and odds of developing seronegative (RF− and CCP−), seropositive (RF+ or CCP+), erosive, and seropositive, erosive RA phenotypes. In separate case only analyses, we assessed the relationships between GRS and age of symptom onset.

In 542 RA cases, 317 (58%) were seropositive, 163 (30%) had erosions and 105 (19%) were seropositive with erosions. Comparing the highest GRS risk group to the median group, we found an OR of 1.2 (95% CI = 0.8–2.1) for seronegative RA, 3.0 (95% CI = 1.9–4.7) for seropositive RA, 3.2 (95% CI = 1.8–5.6) for erosive RA, and 7.6 (95% CI = 3.6–16.3) for seropositive, erosive RA. No significant relationship was seen between GRS and age of onset.

**Conclusions/Significance:**

Results suggest that seronegative and seropositive/erosive RA have different genetic architecture and support the importance of considering RA phenotypes in RA genetic studies.

## Introduction

Rheumatoid arthritis (RA) is a chronic systemic inflammatory disease that often leads to disability from joint damage and inflammation. Although RA is uncommon, with a worldwide prevalence of approximately 1%, it has a large economic and societal cost, primarily in terms of work-related disability [1]. Bony destruction, or erosions, are associated with work disability [2] and lower functional status [3], [4] and thus with a more debilitating disease phenotype. Before the more widespread use of biologics, the incidence of erosions in RA patients was ∼70% within the first 3 years after diagnosis [5]. However, with more advanced treatment the prevalence of erosions has decreased [6], [7].

Recently in genetic studies, RA has been divided into two phenotypes defined by presence or absence of serologic factors. Originally the subdivision was based on rheumatoid factor (RF), but more recently antibodies to cyclic citrullinated proteins (anti-CCP) have been used to define the two subtypes [8], [9], [10], [11]. Both RF and anti-CCP positivity have been linked to more severe disease features and outcomes [12], [13], [14], [15]. Presence of RF has also been shown to be a major predictor of both development and severity of joint erosions [16], [17], [18], [19]. Based on these previous results, we defined RA on a continuum of disease severity ranging from seronegative RA (least severe phenotype) to seropositive RA or erosive RA (more severe phenotypes) and finally to seropositive, erosive RA (most severe phenotype).

In addition to erosions and serologic status, age at onset of disease has been associated with RA outcomes, although the results have been varied, with older disease onset predicting worse outcomes in some [20], [21], [22], [23] and milder outcomes in other studies [22], [24]. Specifically, Bukhari and colleagues showed that those with older age of RA onset had higher odds of developing erosive disease and a worse severity of erosions as compared to those with onset <50 years old [25]. Moreover, Pease et.al. reported a slight increase in odds of erosions (although non-significant) for those with onset 65 years or older, but in contrast also found a 3 fold increase in odds of RA disease remission for the same age group [22]. Earlier studies have also shown a lower prevalence of HLA-DR4, the major genetic risk factor for RA, in patients with later onset of RA, although not always significant [26], [27], [28], [29]. Similar to serologic status, these and other studies have suggested that RA could potentially be divided into 2 subsets defined by earlier versus later age at onset [22], [26], [27], [28], [30].

## Methods

### Objectives

Karlson et al. showed that a weighted Genetic Risk Score (GRS) with 22 RA risk alleles showed a good discrimination between seropositive RA and controls. The addition of a weighted GRS score comprised of validated genetic risk factors showed improved discrimination when compared to a model with just clinical risk factors alone [31]. We extend this analysis in two ways, first, by adding the newly validated RA risk alleles to the GRS [32], [33], [34], [35] and second, by assessing the GRS in relation to the more specific phenotypes of RA along the severity continuum, including erosive status, seropositivity and age at first symptom onset of RA. We will show that the GRS is most applicable for the more extreme RA phenotypes defined by seropositive and erosive status, and consequently that these phenotypes have a different genetic architecture than the seronegative and non-erosive forms of the disease.

### Participants

The Nurses' Health Study is a prospective cohort which enrolled 121,700 female nurses aged 30 to 55 years throughout the US in 1976. Of those, 32,826 (27%) participants provided blood samples for future studies and an additional 33,040 (27%) provided buccal cell samples for a total of 65,866 (54%) samples with available DNA. A similar prospective cohort, Nurses' Health Study II enrolled 116,609 female nurses aged 25 to 42 years in 1989, of which 29,611 (25%) provided blood samples for future studies. For these analyses the two cohorts will be combined and referred to as ‘NHS’.

### Ethics

All aspects of this study were approved by the Partners Human Research Committee, the Institutional Review Board of Partners Research Management. Three types of written informed consent were acquired for these studies. First, for questionnaires, NHS cohort participants were consented by paper at baseline in 1976 for repeated surveys and NHSII cohort participants were consented by paper at baseline in 1989 for repeated surveys. Second, for the sub-cohorts who contributed blood for the genetic analysis, the NHS participants were consented by paper in 1989 at blood draw and the NHS2 participants were consented in 1997 at blood draw. Finally, all RA self-reported cases signed informed consent to release medical records for review.

### Phenotypic Rheumatoid Arthritis

A staged screening method was used to confirm cases of RA in the NHS cohort. A connective tissue disease (CTD) screening questionnaire was used to screen all self-reported cases for RA symptoms, followed by chart validation by two board-certified rheumatologists [36]. Four phenotypes of RA were defined using rheumatoid factor (RF) and/or CCP positivity and presence of radiographic changes/erosions. Rheumatoid factor was determined by chart review. Second generation CCP assays were performed among available pre-diagnosis or post-diagnosis blood samples for a subset of cases (n = 273 cases) as previously described [37], all others were obtained by chart reviews since the mid-2000's when the test became widely available. Erosions were determined by chart review [37]. The four RA phenotypes of primary interest included: 1) seronegative RA (both RF and CCP negative, n = 225, in supplemental); 2) seropositive RA (either RF+ or CCP+ or both, n = 317); 3) Erosive RA (presence of erosions, n = 163); and 4) seropositive, erosive RA (presence of erosions AND in group 2, n = 105). In addition, we also examined all RA (n = 542) and non-erosive RA (n = 379) as a supplemental analysis. The groups are not mutually exclusive, i.e. cases in the seropositive, erosive RA group are also included in the seropositive RA and the erosive RA groups. Age at RA onset was determined from chart reviews as either age at onset of RA symptoms, if available, or age at RA diagnosis.

### Genotyping

Low resolution *HLA-DRB1* genotyping was performed using PCR with sequence-specific primers (SSP) using OLERUP SSP kits (Qiagen, West Chester, Pennsylvania, USA), as previously described [38]. For samples with positive two-digit human leucocyte antigen (HLA) signals, SSP were used for high- resolution four-digit allele detection of DRB1*0401, *0404, *0405, *0408, *0101, *0102, *09 and *1001. All non-MHC risk alleles were genotyped using iPlex (Sequenom, San Diego, California, USA) at the Broad Institute, as previously described [33]. All SNP had call rates greater than 95% and Hardy–Weinberg equilibrium p values greater than 0.01.

### Creation of Genetic Risk Score (GRS)

Thirty-nine validated risk alleles for RA are combined to form a continuous GRS. This GRS is a weighted combination of 8 HLA-DRB1 ‘shared epitope’ (HLA-SE) alleles and 31 non-MHC risk alleles. Each allele is weighted by the natural log of the published OR and then summed over 39 alleles, as previously described [31], [39], [40]. Using a weighted risk score is important in this case since both PTPN22 and the HLA-SE have a stronger predictive relationship with RA as compared to the more recently discovered SNPs. The list of alleles and weights are presented in Supplemental Table 1. We evaluated the linkage disequilibrium (LD) structure of the risk alleles using HapMap release 22 and found little evidence of LD (largest R^2^ = 0.06) suggesting that LD has little to no affect on the variance of the GRS.

The ORs and weights for the HLA-SE alleles are from a meta-analysis of all published studies [41]. Odds ratios and weights for 5 out of the 31 non-MHC SNPs were taken from extensively replicated SNPs from published studies. These include *PTPN22* (rs2476601) [42], *TRAF1-C5* (rs3761847) [43], *STAT4* (rs7574865) [44], *TNFAIP3* (rs17066662, in LD with rs1099194, r^2^ = 1.0) [45] and *TNFAIP3* (rs6920220) [45]. Odds ratios and weights for 9 of the 31 non-MHC alleles were taken from a meta-analysis of GWAS data for 3,393 cases and 12,462 controls with replication in 3,929 seropositive RA cases and 5,807 matched controls by Raychaudhuri et al, [33]. To avoid over-estimation of the true effect size we used the ORs from the replication phase of the study [32]. These SNPs include *CD40* (rs4810485), *CCL21* (rs2812378), *CTLA4* (rs3087243), *PADI4* (rs2240340), *CDK6* (rs42041), *TNFRSF14* (rs3890745), *PRKCQ* (rs4750316), *KIF5A* (rs1678542), and *4q27 (IL2/IL21)* (rs6822844) [33]. The ORs and weights for 7 risk alleles were selected from the joint analysis from Raychaudhuri, et al [34] and were identified as functionally related to known RA risk loci by GRAIL, a bioinformatics analysis that identifies connections among genes in published abstracts. These include *PTPRC* (rs10919563), *CD2* (rs11586238), *CD28* (rs1980422), *TAGAP* (rs394581), *RAG1* (rs540386), *PRDM1* (rs548234), and *FCGR2A* (rs7552317) [34]. Finally, 10 SNPs were selected from the final combined analysis from a genome-wide association study (GWAS) meta-analysis of 5,539 autoantibody positive RA cases and 20,169 controls of European descent, followed by replication in an independent set of 6,768 RA cases and 8,806 controls [35]. These include SPRED2 (rs934734), *ANKRD55/IL6ST* (rs6859219), *C5orf13/GIN1* (rs26232), *PXK* (rs13315591), RBPJ (rs874040), *CCR6* (rs3093023), *IRF5* (rs10488631), *AAF3* (rs11676922), CCL21 (rs951005) and *IL2RA* (rs706778).

The continuous GRS score was then divided into 7 sub-groups. The thresholds for the groups were based on the Gaussian distribution in the controls. A more detailed description of methods is published elsewhere [31]. Briefly, dividing our score into 7 categories provided the most robust distribution, allowing us to parse out the highest and lowest risk groups while ensuring that there were sufficient numbers of cases and controls in these extreme categories of interest.

### Study Sample Filtering

Each confirmed RA case was matched to one healthy control by cohort (NHS or NHSII), year of birth, menopausal status, and post-menopausal hormone use. Our initial nested case-control dataset consisted of 585 RA cases and 585 matched controls. To reduce the potential for population stratification we limited our analysis to self-reported Caucasian women, resulting in 564 RA cases and 571 controls. Since the HLA alleles have a large weight in the GRS we dropped any participant missing HLA data. Among the 564 RA cases, 22 (4%) were missing HLA, and among the 571 healthy controls, 20 (4%) were missing HLA. This left us with 542 RA cases and 551 healthy controls. For anyone missing other SNPs, we assigned them a value equal to the expected value (2*risk allele frequency defined in cases or controls separately).

### Epidemiological Covariates

Smoking is the strongest environmental factor linked with RA, and its population attributable risk is 25% for all RA and 35% for seropositive RA [46], [47], [48]. Prospective, biennial questionnaires were used to collect covariate information from all NHS subjects. The questions include inquiries regarding diseases, lifestyle and health practices. Lifetime history of smoking was collected at the baseline questionnaire and data concerning current smoking status and number of cigarettes smoked per day were updated in each two year questionnaire cycle. Pack-years were calculated as number of packs per day smoked times number of years of smoking using the questionnaire cycle prior to the date of RA diagnosis or index date for matched controls.

### Statistical Analysis

Demographic characteristics of the cohorts are described using means and standard deviations for continuous variables and frequency and proportions for categorical variables. Logistic regression analysis was used to calculate the odds of a phenotypic RA for each GRS risk group as compared to the median group (group 4). The odds of phenotypic RA for the most extreme risk group (group 7) as compared to the least extreme risk group (group 1) was calculated using an ordinal model that takes into account all the data in all the groups. A test for linear trend across all seven groups was performed using logistic regression, with each group equaling the median GRS level in that group. The discriminatory ability of the GRS to define case group vs. control group at different combinations of sensitivity and specificity was assessed using a Receiver Operating Characteristic (ROC) curve and computing the Area Under Curve (AUC). Finally, Pearson correlation coefficients were used to compare continuous GRS and age at RA symptom onset and Analysis of Variance (ANOVA) was used to calculate the mean age at RA symptom onset for each GRS risk group. Models were adjusted for year of birth and pack-years of smoking. All analyses were performed on SAS Version 9.1 (SAS Institute, Cary, NC).

## Results

### Subjects

Five hundred and forty two RA cases were identified with a mean age at RA symptom onset of 56 (SD, 11). Of these, 317 (58%) were seropositive, 163 (30%) had evidence of erosions and 105 (19%) had seropositive, erosive RA. Five hundred and fifty-one controls were selected among NHS participants who gave a blood or buccal cell sample. The mean age at time of blood sample was 55 (SD, 8) years for cases and 56 (SD, 8) years for controls. Demographic information for cases and controls are presented in Table 1.

**Table 1:** Characteristics of RA cases and controls in the Nurses' Health Study.

	RA cases (n = 542)	Controls (n = 551)
Age, mean (SD)^a^	55.3 (±8.1)	55.5 (±7.9)
Current or past smoker, n (%)	330 (62%)	309 (56%)
Pack-years among smokers, mean (SD)	25.0 (±18.0)	22.7 (±20.9)
**RA features**		
Mean age at symptom onset, mean (SD)	55.7 (±10.8)	-
Mean age at diagnosis, mean (SD)	56.6 (±10.2)	-
Rheumatoid nodules, n (%)	70 (13%)	-
Rheumatoid factor positive, n (%)	303 (56%)	-
Anti-CCP^2^ positive, n (%)	112 (34%)	-
**Seropositive**, n (%)	317 (58%)	-
**Radiographic changes, erosions**, n (%)	163 (30%)	-
**Seropositive and erosions**, n (%)	105 (19%)	

a Age at blood draw for blood samples (n = 328 cases, n = 334 controls),

2 Cyclic citrullinated protein antibodies assayed in subset of NHS cases (n = 327) with stored blood samples at collected at different points with respect to RA onset, up to 12 years prior to onset or after diagnosis.

### Relationship between Risk Alleles and Seropositive RA

Bivariate associations between single risk alleles and odds of seropositive RA in the NHS data are presented in Supplemental Table S1. In most cases the direction of the association in the NHS data and the published ORs is the same, although most confidence intervals cross the null value of 1.0. This is to be expected since the individual effect sizes are small, and thus we do not have the power to see significant bivariate associations.

### GRS and Odds of Phenotypic RA

**Figure 1:**
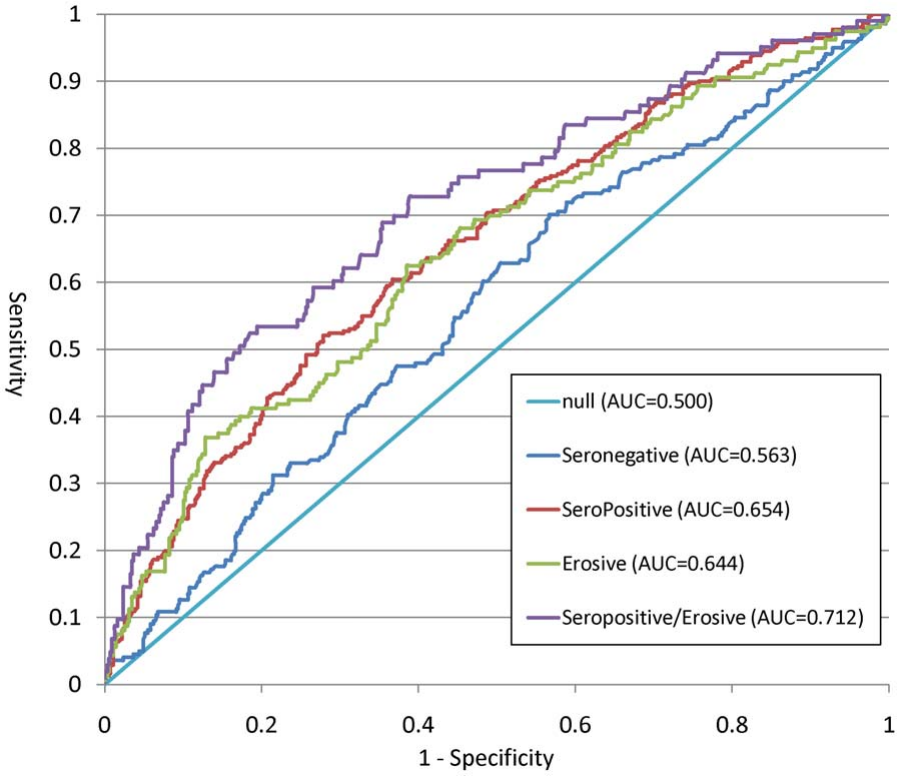
Receiver Operating Characteristic (ROC) curve for 4 phenotypes of RA.

The results of the association analysis of three of the outcomes, seropositive RA, erosive RA and seropositive, erosive RA are presented in Table 2. The additional outcomes, all RA, seronegative RA and non-erosive RA are presented in Supplemental Table S1. Group 4 (the median level of risk) was used as the referent group in this analysis. Those with a GRS in group 7 had a significantly increased odds of seropositive RA (OR = 3.0; 95%CI 1.9–4.7), erosive RA (OR = 3.2; 95%CI 1.8–5.6) and seropositive/erosive RA (OR = 7.6; 95%CI 3.6–16.3), with the highest increased odds being for seropositive/erosive RA. Those in top GRS group (group 7) had no significant increase in odds of seronegative RA (OR = 1.2; 95%CI 0.8–2.1) (Supplementary Table S2). The discrimination ability of the model, measured as AUC, for predicting seronegative RA was 0.563, only slightly above the null value of 0.50 (a null model would have an AUC of 0.500, whereas a perfect model would have an AUC of 1.0). The other 3 phenotypes of RA showed better discrimination with AUCs of 0.654, 0.644 and 0.712 for seropositive RA, erosive RA and seropositive, erosive RA respectively. The ROC curves for 4 outcomes, seronegative, seropositive, erosive and seropositive, erosive RA are represented in **Figure 1**. Since the outcomes vary across the models we cannot directly compare the AUCs using the known methods [49].

**Table 2:** Weighted GRS groups and odd ratios of seronegative, Seropositive, Erosive and Seropositive/Erosive RA in NHS.

GRS39 Group	controls (n = 551)	Seropositive RA (n = 317)		Erosive RA (n = 163)		Sero+, Erosive RA (n = 105)	
	n (%)	n (%)	OR^a^ (95% CI)	n (%)	OR^a^ (95% CI)	n (%)	OR^a^ (95% CI)
**1**	48 (9%)	11 (3%)	0.5 (0.2–1.1)	8 (5%)	0.7 (0.3–1.6)	4 (4%)	1.0 (0.3–3.2)
**2**	84 (15%)	17 (5%)	0.4 (0.2–0.8)	9 (6%)	0.4 (0.2–1.0)	2 (2%)	0.3 (0.1–1.3)
**3**	107 (19%)	57 (18%)	1.2 (0.7–1.8)	27 (17%)	1.0 (0.5–1.8)	19 (18%)	1.9 (0.8–4.3)
**4**	114 (21%)	55 (17%)	1.0 (ref)	28 (17%)	1.0 (ref)	10 (10%)	1.0 (ref)
**5**	84 (15%)	49 (15%)	1.3 (0.8–2.1)	23 (14%)	1.2 (0.6–2.2)	15 (14%)	2.2 (0.9–5.1)
**6**	50 (9%)	40 (13%)	1.7 (1.0–2.9)	19 (12%)	1.5 (0.8–3.0)	14 (13%)	3.4 (1.4–8.1)
**7**	64 (12%)	88 (28%)	3.0 (1.9–4.7)	49 (30%)	3.2 (1.8–5.6)	41 (39%)	7.6 (3.6–16.3)
**p-value** b			1.7×10^−12^		5.6×10^−9^		5.0×10^−12^
**7 vs. 1** c			5.7 (3.5–9.3)		4.9 (3.2–10.8)		14.2 (6.5–30.9)
**AUC**			AUC = 0.654		AUC = 0.644		AUC = 0.712

a adjusted for year of birth and pack-years of smoking;

b for linear trend, using an ordinal model;

c Based on an ordinal model.

In the ordinal model, which takes into account all the data in all the groups, we see a significant increase odds of seropositive RA (OR = 5.7; 95%CI 3.5–9.3), erosive RA (OR = 4.9; 95%CI 3.2–10.8) and seropositive/erosive RA (OR = 14.2; 95%CI 6.5–30.9) for group 7 as compared to group 1 (Table 2). In addition, we see an increased odds of seronegative RA (OR = 2.0; 95%CI 1.2–3.5) for those with a GRS in the top group (group 7) compared to the lowest risk group (group 1) (Supplementary Table S2). Strongly significant linear trends were seen in the seropositive, erosive and seropositive/erosive RA case groups, with all p-values<0.0001 (Table 2). For seronegative RA the p for trend was 0.007 (Supplementary Table S2).

### Association between GRS and Age at RA Symptom Onset

The results for the association between the GRS and age at RA symptom onset are presented in Table 3 and Supplementary Table S3. The adjusted mean ages at RA symptom onset were not significantly different among the 7 GRS groups for any of the phenotype subgroups (p>0.05 for all). The correlations between continuous GRS for seropositive RA, erosive RA and seropositive, erosive RA all were negative (−0.09, −0.08, −0.11 respectively) indicating that the larger the GRS, the younger the age at first symptom; however none of these correlations were significant.

**Table 3:** Relationship between weighted GRS as groups and as continuous and age at RA symptom start.

	Mean Age^a^ (95% CI)		
GRS39 Group	Seropositive (n = 317)	Erosive RA (n = 163)	Sero+ Erosive RA (n = 105)
**1**	51.1 (46.5–55.7)	52.3 (46.5–58.1)	42.3 (37.6–53.0)
**2**	57.4 (53.7–61.2)	55.1 (49.6– 60.6)	55.8 (44.8–66.8)
**3**	55.3 (53.2–57.4)	54.5 (51.2–57.8)	56.3 (52.6–60.1)
**4**	56.8 (54.7–58.9)	54.8 (51.7–58.0)	53.2 (48.1–58.2)
**5**	54.8 (52.6–57.0)	51.5 (48.0–55.0)	49.9 (45.7–54.1)
**6**	53.5 (51.0–56.0)	51.7 (47.8–55.6)	50.6 (46.5–54.8)
**7**	55.2 (53.5–56.8)	54.0 (51.6–56.3)	52.7 (50.2–55.2)
**total**	55.3 (54.1–56.4)	53.5 (51.9–55.2)	52.6 (50.6–54.5)
**ρ** b	−0.090	−0.078	−0.109
**p-value**	0.109	0.325	0.268

a adjusted for year of birth and pack-years of smoking,

b ρ = Pearson correlation coefficient comparing continuous age at RA symptom onset and continuous GRS.

## Discussion

We found that a weighted genetic risk score was associated with development of seropositive RA, erosive RA and seropositive, erosive RA phenotypes. Although there was a significant linear trend with a continuous GRS39 measure predicting seronegative RA, with the exception of group 7 compared to group 1, there was no significant relationship when the score was divided into groups. In contrast, we found a strong and significant association between both continuous and grouped GRS39 and the erosive and/or seropositive phenotypes. Subjects with the highest GRS score (group 7) had a 3.2 times increase of odds of erosive RA as compared to the median group. This odds ratio increased to 7.6 when limiting the phenotype to those with seropositive, erosive RA. We observed similar results when comparing extreme GRS scores (group 7 vs. group 1), where we found a 5 times increased odds of erosive RA and a 14 times increased odds for seropositive, erosive RA. This suggests that the GRS has a stronger association with the more severe phenotype; however narrowing the phenotype definition resulted in a widened confidence interval. Thus, although we detected a stronger effect size (i.e. larger OR), there was also greater variability in the association, most likely due to the small sample size in this group.

One interesting result is the association between the GRS with 39 risk alleles and seropositive RA. We found that group 7 had a 3.0 times increased odds of seropositive RA as compared to group 4. This is similar to the 2.9 times increased odds found by Karlson et al [31] with the GRS based on 22 risk alleles. In addition, we observed a similar increase in ORs in the ordinal model when comparing group 7 to group 1, where the OR was 6.3 (from Karlson et al, with 22 risk alleles) and 5.7 in our analysis that included 17 additional risk alleles. Similarly, the combination of risk alleles also displayed a good ability to discriminate between an RA case and control when the case is defined as seropositive RA, erosive RA or seropositive, erosive RA. However, the GRS showed very little, if any, ability to discriminate between seronegative RA and controls with an AUC of 0.563. When we compare the seropositive RA model with the 39 alleles to the one from Karlson et al. with 22 alleles we see no improvement from 0.660 (GRS22) to 0.654 (GRS39). This suggests that the addition of these 17 newly discovered RA alleles, whose individual ORs range from 1.10 to 1.23, does not improve the predictive ability of the GRS. As genetic discoveries progress with next generation sequencing, it is likely that cumulative GRS will improve in its predictive ability.

Our results for seronegative RA should be viewed in the context of prior research. The loci used in the GRS were discovered and the weights determined using only studies that include seropositive RA cases. Although there have been a few genetic markers that have associated with an increase risk of seronegative RA, such as HLA-DR1*03 [50], HLA-DR3 [51], and allelic forms of *DCIR* [52] and *IRF5* [53], there may be as yet undiscovered loci that predict the seronegative RA phenotype. In a dataset containing 1500 cases/1500 controls, Kurreeman et al [54] demonstrated that a GRS based on 28 non-HLA risk alleles was associated with seronegative RA with an AUC of 0.55 and a p-value for a linear association of 0.0008 also suggesting only a very modest association for these risk alleles with the seronegative phenotype.

It has been shown that the HLA-SE is strongly associated with both RF status and presence of anti-CCP antibodies [9], [55], [56]. More specifically, anti-CCP antibodies play a vital role in the causal pathway between HLA-SE and erosions [29]. This is one explanation of the results demonstrating that GRS39 performs similarly when using erosive status to define severe disease, rather than seropositivity. In addition, the observation of an AUC of 0.712 for GRS39 identifying seropositive, erosive RA cases suggests that a narrower definition of RA leads to better discriminative ability. This lends support to the argument that RA falls along a severity continuum starting with seronegative as least severe and leading to seropositive, erosive RA as most severe.

We found that earlier age at onset of RA may potentially be associated with increased GRS. While the correlations were weak and not statistically significant, this does suggest that perhaps those with earlier age at RA onset have a higher “load” of genetic risk factors than those with later onset. Previous studies have shown an earlier age of diagnosis of RA both for those having any HLA-SE compared to none [26], [27], [28], and for any *PTPN22* T allele compared to CCP [29]. Since both HLA and *PTPN22* have a strong influence on the GRS score, this may be one explanation for the inverse relationship between the GRS and age at onset. The strongest effects that we detected for GRS and age at onset were with the seronegative and seropositive phenotypes. With this number of subjects, we had 37% and 35% statistical power to detect a significant ρ of 0.11 in seronegative and a ρ of −0.09 in seropositive RA. It is possible with more subjects in all phenotype groups we might have been able to detect significant relationships.

One limitation of our study is that we only have anti-CCP status tested at one time point, either up to 12 years prior to time of RA diagnosis or after diagnosis for the subset of cases without blood sample collected. The lack of information for anti-CCP results in the medical records due to the recent development of this test limits our ability to study anti-CCP results after diagnosis in all cases. We have not systematically collected outcome data after diagnosis of RA in this cohort, thus we do not know if some of the subjects defined as seronegative at diagnosis will later go on to convert to seropositive. This could lead to misclassification bias, with some truly seropositive RA subjects being misclassified as seronegative, which would bias us away from the null in the analysis. However, as we have found only modest associations within the seronegative group we do not believe that this has affected our analysis. This is also the case with erosive disease status, which based on chart data included notes ranging from the date of diagnosis where subjects have not had time to develop erosions to many years of follow-up. Another possible limitation to this study is the lack of data to test for population stratification. However, a subset of this sample (437 RA cases and 437 controls) [38] was genotyped for the lactase gene (rs4988235), known to exhibit substantial variation in allele frequency from Northern to Southern Europe [57], [58]. No significant differences were found between cases and controls, arguing strongly against any significant population stratification in this dataset.

In summary, many arguments have been made in the last few years for subdividing RA into different phenotypes [8], [9], [10], [11]. The analyses here add credence to these arguments. We demonstrate different genetic associations for the different RA sub-types, with only a modest relationship seen in the least severe phenotype, seronegative and the strongest relationship seen with the most severe phenotype, seropositive, erosive RA. This suggests that seropositive RA has a different underlying genetic basis than seronegative RA and thus, in future research, studying the two phenotypes separately would lead to greater understanding of the genetic and functional make-up of the disease.

## Supporting Information

Table S1 **Genotype frequencies and association with seropositive RA in for 39 RA risk alleles.**
(DOCX)

Table S2 **Weighted GRS groups and odd ratios of All, seronegative and non-Erosive RA in NHS.**
(DOCX)

Table S3 **Relationship between weighted GRS as groups and as continuous and age at RA symptom start.**
(DOCX)
