# Supplementary material for: Genetic Risk Score Predicting Risk of Rheumatoid Arthritis Phenotypes and Age of Symptom Onset
Source: PLoS One. 2011 Sep 12;6(9):e24380. doi: 10.1371/journal.pone.0024380 (PMC3171415; doi:10.1371/journal.pone.0024380)
Supplement: Table S1 — Genotype frequencies and association with seropositive RA in for 39 RA risk alleles. (DOCX) [file pone.0024380.s001.docx]

Supplemental Table S1: Genotype frequencies and association with seropositive RA in for 39 RA risk alleles

| **Loci** | **SNP** | **Risk Allele** | **Published OR^b^** | **Weight^c^** | **Cases RAF^d^** | **Controls RAF^d^** | **OR (95% CI)** |
| --- | --- | --- | --- | --- | --- | --- | --- |
| DRB*0401 | SE | SE | 3.30 | 1.194 | 0.14 | 0.09 | 1.57 (1.21 - 2.04) |
| DRB*0404 | SE | SE | 1.85 | 0.615 | 0.05 | 0.04 | 1.47 (0.97 - 2.23) |
| DRB*0405 | SE | SE | 3.84 | 1.345 | 0.01 | 0.00 | 3.78 (1.05 - 13.64) |
| DRB*0408 | SE | SE | 1.04 | 0.039 | 0.01 | 0.01 | 1.19 (0.40 - 3.56) |
| DRB*0101 | SE | SE | 1.60 | 0.470 | 0.09 | 0.06 | 1.45 (1.06 - 1.98) |
| DRB*0102 | SE | SE | 1.10 | 0.095 | 0.01 | 0.01 | 1.15 (0.44 - 2.99) |
| DRB*1001 | SE | SE | 2.35 | 0.854 | 0.01 | 0.01 | 1.54 (0.62 - 3.79) |
| DRB*09 | SE | SE | 1.48 | 0.392 | 0.01 | 0.01 | 1.28 (0.50 - 3.25) |
| PTPN22 | rs2476601 | T | 1.75 | 0.560 | 0.13 | 0.09 | 1.45 (1.11 - 1.90) |
| TRAF1-C5 | rs3761847 | G | 1.32 | 0.278 | 0.44 | 0.42 | 1.09 (0.92 - 1.30) |
| STAT4 | rs7574865 | T | 1.27 | 0.239 | 0.23 | 0.20 | 1.18 (0.96 - 1.45) |
| TNFAIP3 | rs17066662 | C^a^ | 1.33 | 0.285 | 0.74 | 0.73 | 1.06 (0.88 - 1.28) |
| TNFAIP3 | rs6920220 | A | 1.22 | 0.199 | 0.21 | 0.21 | 0.97 (0.79 - 1.19) |
| CD40 | rs4810485 | G^a^ | 1.15 | 0.140 | 0.77 | 0.75 | 1.11 (0.91 - 1.36) |
| CCL21 | rs2812378 | C | 1.12 | 0.113 | 0.36 | 0.34 | 1.13 (0.95 - 1.35) |
| CTLA4 | rs3087243 | G^a^ | 1.11 | 0.104 | 0.53 | 0.53 | 1.01 (0.85 - 1.19) |
| PADI4 | rs2240340 | A | 1.02 | 0.020 | 0.43 | 0.40 | 1.12 (0.94 - 1.32) |
| CDK6 | rs42041 | G | 1.11 | 0.104 | 0.28 | 0.24 | 1.22 (1.00 - 1.48) |
| TNFRSF14 | rs3890745 | T^a^ | 1.12 | 0.113 | 0.68 | 0.68 | 1.00 (0.84 - 1.20) |
| PRKCQ | rs4750316 | C^a^ | 1.14 | 0.131 | 0.83 | 0.81 | 1.13 (0.91 - 1.40) |
| KIF5A | rs1678542 | G^a^ | 1.12 | 0.113 | 0.65 | 0.64 | 1.04 (0.87 - 1.25) |
| IL2/IL21 | rs6822844 | G^a^ | 1.09 | 0.086 | 0.83 | 0.84 | 0.95 (0.76 - 1.20) |
| PTPRC | rs10919563 | G^a^ | 1.14 | 0.131 | 0.88 | 0.87 | 1.05 (0.82 - 1.36) |
| CD2 | rs11586238 | G | 1.12 | 0.113 | 0.26 | 0.25 | 1.04 (0.85 - 1.27) |
| CD28 | rs1980422 | C | 1.12 | 0.113 | 0.25 | 0.26 | 0.95 (0.78 - 1.16) |
| TAGAP | rs394581 | T^a^ | 1.10 | 0.095 | 0.71 | 0.73 | 0.88 (0.73 - 1.07) |
| RAG1 | rs540386 | C^a^ | 1.11 | 0.104 | 0.89 | 0.85 | 1.42 (1.10 - 1.84) |
| PRDM1 | rs548234 | C | 1.10 | 0.095 | 0.32 | 0.29 | 1.17 (0.97 - 1.42) |
| FCGR2A | rs7552317 | T | 1.12 | 0.113 | 0.15 | 0.14 | 1.06 (0.83 - 1.37) |
| SPRED2 | rs934734 | G | 1.13 | 0.122 | 0.48 | 0.50 | 0.90 (0.76 – 1.06) |
| ANKRD55, IL6ST | rs6859219 | C^a^ | 1.23 | 0.207 | 0.80 | 0.77 | 1.19 (0.97 - 1.48) |
| C5orf13, GIN1 | rs26232 | C^a^ | 1.11 | 0.104 | 0.69 | 0.68 | 1.04 (0.86 - 1.25) |
| PXK | rs13315591 | C | 1.20 | 0.182 | 0.07 | 0.06 | 1.17 (0.83 - 1.63) |
| RBPJ | rs874040 | C | 1.16 | 0.148 | 0.32 | 0.32 | 0.97 (0.81 – 1.16) |
| CCR6 | rs3093023 | A | 1.12 | 0.113 | 0.43 | 0.43 | 1.00 (0.84 - 1.18) |
| IRF5 | rs10488631 | C | 1.21 | 0.191 | 0.14 | 0.12 | 1.16 (0.89 - 1.50) |
| AFF3 | rs11676922 | T | 1.14 | 0.131 | 0.47 | 0.46 | 1.06 (0.90 - 1.26) |
| CCL21 | rs951005 | A | 1.16 | 0.148 | 0.84 | 0.84 | 0.99 (0.79 – 1.24) |
| IL2RA | rs706778 | T | 1.12 | 0.113 | 0.42 | 0.40 | 1.06 (0.90 - 1.26) |

^a^Major allele; ^b^with respect to the risk allele; ^c^natural log of OR; ^d^RAF = Risk Allele Frequency
