# Supplementary material for: Genetic Risk Score Predicting Risk of Rheumatoid Arthritis Phenotypes and Age of Symptom Onset
Source: PLoS One. 2011 Sep 12;6(9):e24380. doi: 10.1371/journal.pone.0024380 (PMC3171415; doi:10.1371/journal.pone.0024380)
Supplement: Table S2 — Weighted GRS groups and odd ratios of All, seronegative and non-Erosive RA in NHS. (DOCX) [file pone.0024380.s002.docx]

Supplemental Table S2: Weighted GRS groups and odd ratios of All, seronegative and non-Erosive RA in NHS

| **GRS39 Group** | **Controls (n=551)** | **All RA (n=542)** | | **Seronegative (n = 225)** | | **non-Erosive RA (n=379)** | |
| --- | --- | --- | --- | --- | --- | --- | --- |
|  | **n (%)** | **n (%)** | **OR^a^ (95% CI)** | **n (%)** | **OR^a^ (95% CI)** | **n (%)** | **OR^a^ (95% CI)** |
| **1** | 48 (9%) | 25 (5%) | 0.56 (0.3 – 1.0) | 14 (6%) | 0.6 (0.3 – 1.1) | 17 (5%) | 0.5 (0.3 – 0.9) |
| **2** | 84 (15%) | 40 (7%) | 0.5 (0.3 – 0.8) | 23 (10%) | 0.5 (0.3 – 0.9) | 31 (8%) | 0.5 (0.3 – 0.8) |
| **3** | 107 (19%) | 94 (17%) | 0.9 (0.6 – 1.3) | 37 (16%) | 0.7 (0.4 – 1.1) | 67 (22%) | 0.9 (0.6 – 1.3) |
| **4** | 114 (21%) | 113 (21%) | 1.0 (ref) | 58 (26%) | 1.0 (ref) | 85 (22%) | 1.0 (ref) |
| **5** | 84 (15%) | 80 (15%) | 1.0 (0.7 – 1.5) | 31 (14%) | 0.7 (0.4 – 1.3) | 57 (15%) | 1.0 (0.6 – 1.5) |
| **6** | 50 (9%) | 60 (11%) | 1.2 (0.8 – 1.9) | 20 (9%) | 0.8 (0.4 – 1.4) | 41 (11%) | 1.1 (0.7 – 1.8) |
| **7** | 64 (12%) | 130 (24%) | 2.1 (1.4 – 3.1) | 42 (19%) | 1.2 (0.8 – 2.1) | 81 (21%) | 1.7 (1.1 – 2.6) |
| **p-value^b^** |  |  | 3.8 x 10^-10^ |  | 0.007 |  | 1.4 x 10^-9^ |
| **7 vs. 1^c^** |  |  | 3.7 (2.1 – 6.6) |  | 2.0 (1.2 – 3.5) |  | 3.3 (1.8 – 6.4) |
| **AUC** |  |  | AUC = 0.616 |  | AUC = 0.563 |  | AUC = 0.606 |

^a^adjusted for year of birth and pack-years of smoking; ^b^ for linear trend, using an ordinal model; ^c^Based on an ordinal model
