# Supplementary material for: Genetic Risk Score Predicting Risk of Rheumatoid Arthritis Phenotypes and Age of Symptom Onset
Source: PLoS One. 2011 Sep 12;6(9):e24380. doi: 10.1371/journal.pone.0024380 (PMC3171415; doi:10.1371/journal.pone.0024380)
Supplement: Table S3 — Relationship between weighted GRS as groups and as continuous and age at RA symptom start. (DOCX) [file pone.0024380.s003.docx]

Supplemental Table S3: Relationship between weighted GRS as groups and as continuous and age at RA symptom start

|  | **Mean Age^a^ (95% CI)** | | |
| --- | --- | --- | --- |
| **GRS39 Group** | **All RA (n=551)** | **Seronegative (n = 225)** | **Non-Erosive (n=379)** |
| **1** | 54.7 (51.4 – 57.9) | 57.7 (53.0 – 62.4) | 55.9 (52.0 – 59.8) |
| **2** | 56.2 (53.7 – 58.8) | 55.7 (52.1 – 59.4) | 56.5 (53.6 – 59.4) |
| **3** | 54.6 (52.9 – 56.3) | 53.6 (50.7 – 56.5) | 54.7 (52.7 – 56.7) |
| **4** | 56.5 (55.0 – 58.1) | 56.3 (54.0 – 58.6) | 57.1 (55.4 – 58.9) |
| **5** | 55.7 (53.8 – 57.5) | 56.8 (53.7 – 60.0) | 57.1 (55.0 – 59.2) |
| **6** | 54.2 (52.0 – 56.3) | 55.8 (51.7 – 59.8) | 55.2 (52.7 – 57.7) |
| **7** | 56.5 (55.0 – 57.9) | 58.9 (56.1 – 61.7) | 58.2 (56.4 – 60.0) |
| **total** | 55.7 (54.8 – 56.7) | 56.4 (54.9 – 58.0) | 56.7 (55.6 – 57.8) |
| **ρ^b^** | -0.010 | 0.111 | 0.038 |
| **p-value** | 0.387 | 0.097 | 0.213 |

^a^adjusted for year of birth and pack-years of smoking, ^b^ρ = Pearson correlation coefficient comparing continuous age at RA symptom onset and continuous GRS.
